# Smoking in adolescents with cleft lip and/or palate: prevalence and associated factors

**DOI:** 10.1590/1984-0462/2023/41/2021316

**Published:** 2023-03-13

**Authors:** Maria Júlia Costa de Souza Villela, Solange de Oliveira Braga Franzolin, Gesiane Cristina Bom, Eliane Alves Motta Cabello dos Santos, Gabriela Fávaro Marques da Cunha, Armando dos Santos Trettene

**Affiliations:** a Universidade de São Paulo, Bauru, SP, Brazil.; b Universidade Nove de Julho, Bauru, SP, Brazil.

**Keywords:** Tobacco, Smoking, Smoking cessation, Adolescent, Cleft palate, Cleft lip, Tabaco, Tabagismo, Hábito de fumar, Adolescente, Fissura palatina, Fenda labial

## Abstract

**Objective:**

To assess the prevalence and factors associated with smoking in adolescents with cleft lip and/or palate.

**Methods:**

This is a cross-sectional study, developed in a Brazilian public and tertiary hospital between November 2018 and August 2019. Adolescents aged between 12 and 19 years old, previously submitted to cheiloplasty and/or palatoplasty surgeries were included. Data collection was carried out through interviews, guided by questionnaires referring to sociodemographic issues, use and factors associated with smoking. For statistical analysis, Fisher’s Exact Test, Chi-square, Student’s t test and bivariate logistic regression were used, all with a significance level of 5% (p≤0.05).

**Results:**

102 adolescents participated. The prevalence of smoking was 20.6% (n=21). Having cleft lip and palate was associated with smoking (p=0.012). The bivariate analysis showed that not dating (p=0.001; OR=0.19) and owning a home (p=0.032; OR=0.33) were configured as protective factors for the act of being a smoker, while believing that smoking facilitates interaction with young people or with the group of friends (p=0.043; OR=2.95), having friends who use alcoholic beverages (p=0.002; OR=8.40), having friends who use drugs, except cigarettes (p=0.002; OR=5.33), use alcoholic beverages (p<0.001; OR=10.67) and sexual initiation (p<0.001; OR=7.00), were associated with being a smoker.

**Conclusions:**

From the knowledge of the profile of adolescents most vulnerable to tobacco use, it is possible to plan and implement educational and preventive actions.

## RESUMO

**Objetivo:**

Avaliar a prevalência e os fatores associados ao tabagismo em adolescentes com fissura de lábio e/ou palato.

**Métodos:**

Estudo de corte transversal, desenvolvido em um hospital público e terciário brasileiro entre novembro de 2018 e agosto de 2019. Foram incluídos adolescentes com idade entre 12 e 19 anos, submetidos previamente às cirurgias de queiloplastia e/ou palatoplastia. A coleta de dados foi realizada por meio de entrevista, guiada por questionários referentes a questões sociodemográficas, uso e fatores associados ao tabagismo. Para a análise estatística, utilizaram-se os testes: exato de Fisher, qui-quadrado, *t* de Student e regressão logística bivariada, todos com nível de significância de 5% (p ≤ 0,05).

**Resultados:**

Participaram 102 adolescentes. A prevalência do tabagismo foi de 20,6% (n=21). Possuir fissura de lábio e palato associou-se ao tabagismo (p=0,012). A análise bivariada mostrou que não namorar (p=0,001; OR=0,19) e possuir habitação própria (p=0,032; OR=0,33) configuraram fatores de proteção para o ato de ser tabagista, enquanto acreditar que fumar facilita a interação com os jovens ou com o grupo de amigos (p=0,043; OR=2,95), ter amigos que usam bebida alcoólica (p=0,002; OR=8,40), ter amigos que usam drogas, exceto o cigarro (p=0,002; OR=5,33), usar bebida alcoólica (p<0,001; OR=10,67) e iniciação sexual (p<0,001; OR=7,00) associaram-se a ser tabagista.

**Conclusões:**

Pelo conhecimento do perfil dos adolescentes mais vulneráveis ao uso do tabaco, é possível planejar e programar ações educativas e preventivas.

## INTRODUCTION

Adolescence is characterized by biopsychosocial changes, greater need for socialization and exacerbation of patterns, especially aesthetic and communication, in which health problems experienced, as well as harmful habits, can perpetuate and affect health in adulthood.^1^ In fact, young people tend to adopt risky behaviors, including smoking, which is the most commonly used licit drug in this age group. Added to this is the worrying fact that smoking initiation is increasingly frequent in adolescence.^2^

The factors associated with this deleterious habit have a multifactorial etiology, including aspects of the social, economic, and family scope. Among them, are: being older, having friends and parents who smoke, easy access to cigarettes, male gender, higher family income, regular family relationship, not having a religion, having a paid activity, early sexual initiation, and exposure to domestic violence.^3,4,5,6,7,8,9,10^ On the other hand, receiving guidance from parents about smoking, not living with smokers, and knowing the harmful effects of electronic cigarettes are identified as protective factors.^7^

Among face malformations, cleft lip and/or palate are the most common, with an incidence in Brazil of 1:650 live births, thus constituting a public health problem. Patients affected by this malformation may present functional, aesthetic, and psychosocial problems. Although initially the functional ones are prevalent, later, especially in adolescence, the aesthetic and psychosocial ones stand out.^11^

It is known that adolescents with clefts are exposed to stigmatization, linked to discrimination and prejudice, with negative repercussions on their social life and on the perception of quality of life, especially in cases of greater anatomical complexity.^12^ However, experiencing these situations is linked to the way in which they will establish their social, family, and cultural relationships.^13^

Although studies on the prevalence and associated factors with smoking in adolescents are available, as far as we know, there are no specific studies with adolescents with cleft lip and/or palate, indicating the relevance of this investigation with an unprecedented approach. Considering the psychosocial vulnerability of adolescents with this malformation, as well as the deleterious effects of smoking, determining its prevalence and factors related to its adherence may support future interventions, in addition to helping to establish public policies for prevention and treatment.

Hence, it was sought to assess the prevalence and factors associated with smoking in adolescents with cleft lip and/or palate.

## METHOD

Cross-sectional study, developed in a public and tertiary hospital, specialized in the care of patients with craniofacial anomalies and related syndromes. It is an institution with 91 beds, maintained with resources from the Unified Health System and *Universidade de São Paulo* and recognized nationally and internationally for its excellent performance in the areas of care, teaching, and research.

The population consisted of adolescents with cleft lip and/or palate who were in outpatient care at the institution. Inclusion criteria were: age between 12 and 19 years, cleft lip and/or palate, and having been previously submitted to primary surgeries to correct the lip (cheiloplasty) and/or the palate (palatoplasty). Adolescents who had syndromes and/or other malformations associated with cleft lip and/or palate were excluded.

Although, according to the World Health Organization, adolescents are aged between 10 and 19 years, it was decided to include in this study those aged between 12 and 19 years, considering that the prevalence of smoking below this age group is very low.^7^ 163 adolescents were invited to participate in the research. Of these, 102 accepted the invite, constituting the sample.

Data collection took place between November 2018 and August 2019, through a sociodemographic questionnaire prepared by the researchers to characterize participants according to the variables: age, gender, race, education, school, marital/affective status, socioeconomic classification, housing, religion, having children, having a paid job, and type of cleft (lip, palate, lip and palate).

In order to assess the prevalence and factors associated with smoking, a questionnaire was constructed based on similar investigations,^7,10^ consisting of questions related to smoking and the act of smoking, as well as thoughts and knowledge about tobacco, exposure smoking, family members and friends who smoke and/or use drugs (except cigarettes), initiation of sexual activity, exposure to domestic violence. and family life.

Data collection was performed exclusively by the main researcher, through an interview, in a private environment, individually, while participants were waiting for outpatient care. Initially, the research objectives were informed and the Informed Consent was presented, as appropriate, followed by the data collection instruments.

A smoker was defined as an adolescent who reported having smoked cigarettes on at least one day in the last thirty days, as recommended by the Center for Disease Prevention and Control and the World Health Organization.^14^ Furthermore, considering that smoking is recognized as a disease associated with nicotine dependence, which, in turn, is present in tobacco-based products, adolescents who used hookah and/or electronic cigarettes were included among smokers.^15,16^

The research was approved by the Ethics Committee for Research on Human Beings of the Institution, CAAE: 98395518.3.0000.5441. All precepts of Resolution No. 466/2012 of the National Health Council were complied with.

Data were organized in the Microsoft Excel 2013^®^ program and, later, transported to the Stata Program, version 13^®^, the software used to process the data. Initially, a descriptive analysis was performed through the distribution of frequencies, mean and standard deviation with a view to characterizing participants. For the bivariate analysis, in order to investigate differences between frequencies, the chi-square and Fisher’s exact tests were used. For the continuous quantitative variable, age, the Student’s *t*-test was used.

Smoking was considered as a dependent variable, and the results were presented in relation to smokers and non-smokers. For the variables identified as significant, the Odds Ratio (OR) estimates and their respective 95% confidence intervals were obtained, considering a level of statistical significance of 5% (p≤0.05).

## RESULTS

The prevalence of smoking among adolescents with cleft lip and/or palate was 20.6% (n=21), with 80.9% of them (n=17) using the hookah, alone or in association with cigarettes and electronic cigarette. The mean age among adolescent smokers was higher compared to non-smokers (16.3 and 14.7 years, respectively; p=0.001) (Table 1).

**Table 1.** Age of smoking and non-smoking adolescents.

Age	Smoking (n=21)	Non-smoking (n=81)	p-value
Mean	16.3	14.7	0.001*
Median	17.0	14.5	
^1st^ quartile	14.5	13.0	
3^rd^ quartile	18.0	16.0	
Standard deviation	2.1	2.0	

*Student’s *t*-test.

As for clinical and sociodemographic variables, among smokers, all were single and without children (100%, n=21). There was still a prevalence of those with cleft lip and palate (76.2%, n=16), males (61.9%, n=13), mixed race/color (38.1%, n=8), who attended public school (85.7%, n=18), who were not dating (57.1%, n=12), belonging to a low social class (66.7%, n=14), living in their own home (57 .1%, n=12), with religion (90.5%, n=19) — of which, 38.1% (n=8) declared to be practitioners — and without an employment relationship (81.0%, n=17) (Table 2).

**Table 2.** Characterization of adolescents in terms of clinical and sociodemographic variables.

Characteristics		Smoking (n=21) n (%)	p-value
Tpe of cleft*	Lip	4 (19.0)	0.012
	Lip and palate	16 (76.2)	
	Palate	1(4.8)	
Gender^†^	Male	13 (61.9)	0.442
	Female	8 (38.1)	
Race/color*	White	7 (33.3)	0.471
	Brown	8 (38.1)	
	Black	3 (14.3)	
	Others	3 (14.3)	
School*	Public	18 (85.7)	0.003
	Private	2 (9.5)	
	Not studying	1 (4.8)	
Affective status^†^	Not dating	12 (57.1)	0.656
	Dating	9 (42.9)	
Social class*	Middle	7 (33.3)	0.164
	Low	14 (66.7)	
Housing^†^	Own	12 (57.1)	0.651
	Rented	9 (42.9)	
Religion*	Yes	19 (90.5)	0.006
	No	2 (9.5)	
Practitioner of the religion^†^	Yes	8 (38.1)	0.532
	No	13 (61.9)	
Work*	Yes	4 (19.0)	0.050
	No	17 (81.0)	

*Fisher’s exact test; ^†^chi-square test,

It was evidenced that having cleft lip and palate (p=0.012), studying in a public school (p=0.003), having a religion (p=0.006), and not working (p=0.050) were significantly associated with smoking (Table 2).

Bivariate analysis showed that not dating (p=0.001; OR=0.19) and having their own home (p=0.032; OR=0.33) configured protective factors for being a smoker (Table 3).

**Table 3.** Factors associated with smoking among adolescents with cleft lip and/or palate.

Characteristics		Smoking	Non-smoking	p-value	OR	95%CI
		n (%)	n (%)			
Type of cleft*	Lip and palate	16 (76.2)	57 (70.4)	0.604	--	--
	Lip or palate	5 (23.8)	24 (29.6)			
Gender*	Male	13 (61.9)	39 (48.1)	0.262	--	--
	Female	8 (38.1)	42 (51.9)			
School^†^	Public	18 (90.0)	68 (89.4)	1.000	--	--
	Private	2 (10.0)	8 (10.5)			
Affective status*	Not dating	12 (57.1)	71 (87.7)	0.001	0.19	0.06–0.56
	Dating	9 (42.9)	10 (12.3)			
Social Class^†^	Middle	4 (22.2)	17 (21.0)	1.000	--	--
	Low	14 (77.8)	64 (79.0)			
Housing*	Own	12 (57.1)	65 (80.2)	0.032	0.33	0.12–0.91
	Rented	9 (42.9)	16 (19.8)			
Religion^†^	Yes	19 (90.5)	73 (90.1)	1.000	--	--
	No	2 (9.5)	8 (9.9)			
Practitioner of the religion*	Yes	8 (40.0)	50 (61.7)	0.085	--	--
	No	12 (60.0)	31 (38.3)			
Work^†^	Yes	4 (19.0)	19 (23.5)	0.786	--	--
	No	17 (81.0)	62 (77.5)			

*chi-square test; ^†^Fisher’s exact test; OR: *odds ratio*, CI: confidence interval.

On the other hand, believing that smoking facilitates interaction with young people or with a group of friends (p=0.043; OR=2.95), having friends who drink alcohol (p=0.002; OR=8.40), who use drugs, except cigarettes (p=0.002; OR=5.33), use of alcohol (p<0.001; OR=10.67) and sexual initiation (p<0.001; OR=7.00) were associated with being a smoker. Participants who did not answer the question were excluded from the statistical analysis (Table 4).

**Table 4.** Factors associated with smoking among adolescents.

Characteristics	Smoking	Non-smoking	p-value	OR	95%CI
	n (%)	n (%)			
Believing that smoking facilitates interaction with young people or with a group of friends*					
Yes	8 (38.1)	14 (17.3)	0.043	2.95	1.03–8.44
No	13 (61.9)	67 (82.7)			
Having friends who drink alcohol^†^					
Yes	19 (90.5)	43 (53.1)	0.002	8.40	1.83–38.42
No	2 (9.5)	38 (46.9)			
Having friends who use drugs, with the exception of cigarettes*					
Yes	8 (38.1)	9 (11.1)	0.002	5.33	1.72–16.54
No	12 (57.1)	72 (88.9)			
Did not respond	1 (4.8)	–			
Use of alcohol*					
Yes	12 (57.1)	9 (11.1)	<0.001	10.67	3.52–32.29
No	9 (42.9)	72 (88.9)			
Sexual initiation*					
Yes	11 (52.4)	11 (13.6)	<0.001	7.00	2.41–20.34
No	10 (47.6)	70 (86.4)			

*chi-square test; ^†^Fisher’s exact test; OR: *odds ratio*, CI: confidence interval.

## DISCUSSION

The prevalence of smoking found in this study was 20.6%. Different investigations showed lower percentages, that is, the hypothesis of the present study was confirmed, in which the smoking rate among adolescents with cleft lip and/or palate was higher compared to those without cleft, according to data presented in the literature, such as among 370 adolescents from the public school system of a municipality in Bahia, whose percentage of smokers was 17.6%.^10^ Another study carried out in the same region showed that, of the 1,170 school adolescents attending the 7^th^ grade, 16.9% consumed tobacco.^5^ Also, among the 798 adolescents, aged between 12 and 19 years old, living in the city of Uruguaiana, in the southern region of Brazil, 13% were smokers.^7^ Even lower percentages were evidenced in other studies.^9,17,18^

However, comparisons are limited by the divergent age groups considered in the studies, the fact that most included only adolescents who attended school, as well as the criteria used to classify smoking.

Among smokers, 80.9% used the hookah, alone or in association with cigarettes and/or electronic cigarettes. The sale of electronic cigarettes is prohibited in Brazil, while the use of hookah is allowed. Both are part of the new attractive wave of tobacco use, with eye-catching and charming devices, which has substantially contributed to the increase in the number of users worldwide, especially among young people, configuring a challenge for professionals and the health system. It is noteworthy that the tobacco used in this device usually has a sweet and pleasant odor and taste, although nicotine concentration is extremely high.^15,16,18^

Regarding the universe of adolescents with cleft lip and/or palate, the use of hookah may be associated with the need for social interaction, since its use is collective, that is, several people use the device simultaneously. Being inserted in social groups and having friends reinforce feelings of acceptance, belonging, and social interaction, in addition to minimizing negative feelings and concerns related to physical appearance.^12,13,19^

In this sense, establishing coping modalities is essential: for example, religiosity and/or spirituality.^12,13,20,21,22^ However, in this study, having a religion was associated with smoking, possibly because, although 90.5% of adolescent smokers reported having a religion, 62% did not practice it.

Not working was also associated with smoking in this study. In general, adolescents do not perform paid activities, particularly those in the age group of this investigation, which was 15.4 years old, and which may explain this finding. It should also be considered that adolescents with cleft lip and/or palate may experience isolation, sadness and loneliness, reasons that are identified as risk factors for drug use, including tobacco.^23^

On the other hand, the literature showed a prevalence of smoking among adolescents who performed paid activities, either due to their low socioeconomic status, or with the aim of earning an income that favors the purchase of cigarettes.^17^ Also, having an older age group and being of the male were associated with smoking, corroborating the findings of other investigations.^3,4,10^

Bivariate analysis indicated that not dating and having their own home were established as protective factors for smoking. Dating favors social interaction, both with peers and in a group, through participation in events, parties or when going to bars, environments that can encourage contact with tobacco.^24^ As for owning a house, it is believed to be linked to socioeconomic aspects. In fact, a multicenter study that included adolescents from 35 European and North American countries showed that adolescents from low-income families had an increased risk of smoking.^2^

In contrast, believing that smoking facilitates interaction with young people or with a group of friends increased the chance of being a smoker by approximately three times, having friends who use alcohol increased it by eight times, those who use drugs, except cigarettes, by five times, use of alcohol by eleven times, and sexual initiation by seven times.

At this stage of life, the power of friendships often exceeds that of the individual, and, in order to be accepted, young people submit to the opinion of others.^25^ Not differing from this reality, the adolescents in this study also presented this characteristic, since having friends who use drugs other than cigarettes was one of the three variables associated with smoking.

Another variable associated with tobacco use referred to using or having friends who use alcohol, pointing out that adolescents with cleft lip and/or palate do not differ from those without clefts, as they also suffer from these harmful influences, which can compromise the intellect and fatally impact their lives.

This finding highlights the social influence on the choices and behaviors established by adolescents, since, in the search for their own identity, they adopt classic adult behaviors, and it is important that family members and friends present themselves as a source of support and inspiration regarding positive references at this stage of life. Therefore, preventing smoking in the early years of adolescence, considering the joint awareness of friends and family, is imperative.^4–6^

In this direction, a study showed that consuming alcohol, using illicit drugs and having smoking friends behaved as risk factors associated with the transition from experimenting with cigarettes to smoking.^26^ Another investigation showed parents and smoking friends as the main influences for smoking.^27^

The prevalence of smoking among male adolescents with cleft lip and/or palate evidenced in this study may be related to the social construction of gender, which educates boys to assume more daring and fearless behaviors, in addition to justifying the association of sexual initiation to tobacco use, since, culturally and socially, male adolescents are encouraged to start their sexual life early.^28,29^ In fact, sexual initiation increased the possibility of being a smoker by about eight times. A similar result was observed in another study, in which this variable, by itself, was associated with smoking in 37.8% of the 143 participants.^10^

Although adolescents with clefts may present difficulties in social interaction and stigmatization, this condition did not prevent or delay sexual initiation, allowing to infer that exposure is independent of official relationships, such as dating, since 57% of smokers reported not dating.

In summary, the prevalence of smoking among adolescents with cleft lip and/or palate was high, especially among those with the most anatomically and functionally complex malformations. Not dating and having their own home were protective factors against smoking, while believing that smoking facilitates interaction with young people or with a group of friends, having friends who use alcohol and drugs, except cigarettes, using alcohol and having started sexual activity were associated with being a smoker.

Finally, it is considered pertinent to point out some limitations of this study, such as the fact that it did not include all the variables that could be associated with smoking among adolescents, as well as the lack of identification of patterns and magnitude related to tobacco. In addition, due to the impossibility of the numerical range initially determined by means of sample calculation, convenience sampling makes it impossible to generalize results, multiple analysis, and internal and external validity. Thus, further investigations with more robust samples and statistical analyses are necessary, in order to expand and consolidate the findings. However, the benefits of this investigation are evident, mainly by strengthening the hypothesis that adolescents with cleft lip and/or palate are more vulnerable to smoking, for which preventive actions should be prioritized in order to minimize the possibilities of being adult smokers and experiencing the numerous disadvantages of this practice.
